# Bio-Repository of DNA in stroke (BRAINS): A study protocol

**DOI:** 10.1186/1471-2350-12-34

**Published:** 2011-03-02

**Authors:** Sunaina Yadav, Renata Schanz, Ankita Maheshwari, Muhammad Saleem Khan, Julia Slark, Ranil de Silva, Paul Bentley, Philippe Froguel, Jaspal Kooner, Padma Shrivastav, Kameshwar Prasad, Pankaj Sharma

**Affiliations:** 1 Imperial College Cerebrovascular Research Unit (ICCRU), Imperial College London, UK; 2 Department of Neurology, All India Institute of Medical Sciences, New Delhi, India; 3 Department of Anatomy, University of Sri Jayewardenepura, Nugegoda, Sri Lanka; 4 Department of Genomics of Common Diseases, Imperial College London, UK; 5 National Heart and Lung Institute, Imperial College London, UK

## Abstract

**Background:**

Stroke is one of the commonest causes of mortality in the world and anticipated to be an increasing burden to the developing world. Stroke has a genetic basis and identifying those genes may not only help us define the mechanisms that cause stroke but also identify novel therapeutic targets. However, large scale highly phenotyped DNA repositories are required in order for this to be achieved.

**Methods:**

The proposed Bio-Repository of DNA in Stroke (BRAINS) will recruit all subtypes of stroke as well as controls from two different continents, Europe and Asia. Subjects recruited from the UK will include stroke patients of European ancestry as well as British South Asians. Stroke subjects from South Asia will be recruited from India and Sri Lanka. South Asian cases will also have control subjects recruited.

**Discussion:**

We describe a study protocol to establish a large and highly characterized stroke biobank in those of European and South Asian descent. With different ethnic populations being recruited, BRAINS has the ability to compare and contrast genetic risk factors between those of differing ancestral descent as well as those who migrate into different environments.

## 1. Background

Evidence shows that stroke incidence rates in developing South Asian countries have increased by more than 100% during the last four decades, while they have decreased by 42% in developed European countries over the same time period [1]. Over a four-decade period, stroke incidence rates increased from 52 per 100,000 person-years (1970-1979) to 117 per 100,000 person-years (2000-2008) and at this rate stroke is expected to become one of the major causes of death in South Asia [1]. While much of this increase may be attributed to changing lifestyles, a high percentage of all strokes occur without any obvious aetiological cause suggesting additional factors that contribute to the stroke risk variability between ethnic groups. The ethnic differences may arise due to varying lifestyles, differences in medical treatment or the inherent genetic makeup.

Dissecting the genetic causes of a complex disorder such as stroke and estimating their relative attributable risks, necessitates a large study cohort in-order to provide sufficient power to the analysis. Herein, we describe a study protocol of two ongoing prospective, multicenter recruitment studies, BRAINS-UK and BRAINS-South Asia which aims to recruit 1500 highly characterised European Caucasian stroke patients from the United Kingdom and 1500 South Asians stroke patients from UK, India and Sri Lanka respectively from participating centres across the three countries. Both case control cohorts will contribute to a combined hypothesis-free genome wide association study (GWAS) approach in identifying novel gene markers of stroke in European Caucasians and South Asians from UK, India and Sri Lanka. BRAINS will be invaluable for future candidate-gene, GWAS and whole genome sequencing studies to identify the genetic associations of all forms of cerebrovascular disease including transient ischemic attacks (TIA), ischemic and hemorrhagic strokes. We aim to gain a much better understanding of the genetic differences between South Asians and the European Caucasian population. BRAINS uses similar definitions of stroke and stroke classification as the successfully completed candidate gene based ISGS and GWAS based SWISS studies [2,3].

South Asians from the Indian sub-continent form the second largest ethnic group in the world and make up some 20% of the world's population whereas Caucasian Europeans constitute about 13%. Both ancestral populations have a genetically stratified structure [4,5] which has led to the wide belief that genetic underpinnings of disease in ethnic populations differ. This is supported by population-based and case-control studies that have demonstrated significant differences in incidence and prevalence in stroke between ethnic groups that are only partly accounted for by differences in environment [6-10]. A number of modifiable risk factors have also been shown to account for the differences between the two populations including age, sex, hypertension, diabetes and prevalence of prior disease [11]. It is recognized that as South Asians migrate out of their native environments and expose themselves to new environmental factors such as diet, their chances of developing vascular disease increase greater than would be expected in the host population [11]. These, along with other epidemiological and molecular observations[12] suggest that genetic effects on stroke may depend upon ethnic background. As the burden of stroke is expected to greatly increase in India over the next decade it is important to characterize these genetic factors, and to compare them with those in European populations. Deciphering the molecular pathways underlying stroke in different populations offers the promise of therapies tailored to genetic (or racial) background, similar to the recognition of racial dependency of anti-hypertension treatment [13,14].

The diagnosis of stroke can be complex with a large number of clinical mimics and no known reliable blood biomarkers or definitive genes to predict the risk of developing stroke or incidence of disease. While the majority of symptomatic strokes can be attributed to modifiable risk factors such as hypertension, age and prior disease [15], these factors do not explain why some individuals are more susceptible to environmental determinants compared to others with the same given risk factors. A 4-fold increase in the risk of stroke in progeny of stroke patients [16,17], clustering of ischemic stroke in families [18] and occurrence of over 10% of haemorrhagic strokes in patients with a prior family history of the same condition [19] lend support to genetic factors being involved in causation of common, 'acquired' strokes, in addition to rarer familial stroke syndromes such as sickle cell disease and CADASIL which have been seen disproportionately in certain racial groups.

Candidate-gene case-control studies have identified multiple genetic polymorphisms associated with stroke. The most consistent associations found are: Methylenetetrahydrofolate Reductase (MTHFR), Factor V Leiden (FVL), Angiotensin-converting enzyme (ACE) and Prothrombin (PT)[20]. Some genes found to be risk factors for one subtype of stroke may be protective towards the other, such as Factor V Leiden a known prothrombotic risk factor for ischemic stroke but likely protective against haemorrhagic stroke [21]. Other studies have implicated genes MTHFR, ACE (I/D) and Apolipoprotein *E *(ApoE) e4 as risk factors for haemorrhagic stroke in European Caucasians whereas Factor V Leiden is shown to be protective [22]. In agreement with the European Caucasian population, these genes have also been implicated as risk factors for ischemic stroke in people of Asian ethnicity (Chinese, Korean and Japanese) [23]. Our group has also identified MTHFR, Factor V Leiden and Prothrombin as genetic risk factors of cerebral venous thrombosis (CVT) in Caucasians adults and children [24] as well as South Asians from India, Pakistan and Bangladesh. Collectively, the above studies help us understand the possible molecular mechanisms underlying stroke and provide us with our best hope for developing population specific drugs to combat this devastating disease.

## 2. Methods and Study Design

### 2A. Ethical consideration

Knowledge of an individual's genetic profile can have adverse effects on their ability to obtain health insurance, employment [25,26] and acceptance in a social group [27]. The BRAINS study meets all ethical standards set by local institutional review boards and has received full institutional ethics approval for the UK and South Asian arms. Written informed consent will be obtained for every proband and control. For intubated patients or those rendered incompetent by stroke, surrogate consent, within review board specifications, will be sought. This will ensure that the BRAINS study is unbiased towards discovery of genetic risk factors for across the entire range of stroke severity. Patient details will be encrypted and patient confidentiality protected. BRAINS investigators who have access to genetic data will be blinded to individual personal identifiers such as names, addresses, phone numbers and email addresses. Investigators who have access to the personal identifiers will be blinded to the genetic data. No subject will be told the result of genetic testing as the individual risk profile in polygenic disorders cannot be accurately measured. All data collection forms will be stored confidentially at the centres from where the individuals were enrolled. Research data and genetic test results will not be placed in the patients clinical notes. Any future project applications to access BRAINS from investigators will be assessed and will have to follow strict MRC ethical guidelines [28]. A future use agreement will be designed according to proposed guidelines [2] to ascertain the appropriate use of BRAINS DNA by an investigative group and maintain transparency in the collaborative efforts.

### 2B. Study population

Patients and spouse or age-matched controls will be screened at the participating clinical centres in the United Kingdom (London, Luton, Birmingham, Leeds, Leicester, Airedale, Bradford, Wolverhampton and Blackburn), India (New Delhi) and Sri Lanka (Colombo). Detailed phenotype data will be collected in a specially designed BRAINS data collection form. Stroke will be confirmed with computed tomography or magnetic resonance imaging and subtyped according to the Trial of ORG10172 in Acute Stroke Treatment (TOAST) classification [29]. Baseline clinical and demographic data will also be collected. Blood samples will be collected in EDTA coated plastic serological tubes from all consenting patients and controls by means of a single venepuncture and DNA extracted from peripheral lymphocytes using standard procedure and stored at -20°C. Following genotyping the genetic data will be merged with phenotype data (environmental risk factors, stroke sub-type, clinical results) for analysis.

### 2B1. Probands (Cases)

***BRAINS-UK ***will recruit 1500 cases of ischemic and haemorrhagic stroke patients > 18 years of age from specialist stroke centres around the UK. ***BRAINS-SA ***will recruit 1500 cases of ischemic and haemorrhagic stroke from specialist stroke centres around the India and Sri Lanka. The project has the advantage of using already extensively investigated patients with stroke. There are likely to be a few sex differences in the overall cohort but any small differences can be adjusted for.

Each patient with suspected stroke will be admitted to the participating centre and evaluated by an in-house neurologist according to institutional patient care guidelines which are based on current international standards of patient care [30,31]. The patient evaluation includes recording patient history (medical, socio-economic and financial) physical examination (BP, pulse rate), CT or magnetic resonance imaging (MR) of the head, and laboratory testing (including lipid profile, CRP, ESR, and Glucose). Exposure to environmental risk factors such as smoking, alcohol and dietary habits will also be recorded. In each participating centre patients seen in inpatient acute stroke services will be reviewed. All probands will be identified using the following inclusion criteria: (1) Patients should be aged > 18 years at the time of enrolment; (2) Diagnosis of haemorrhagic or ischemic stroke using WHO guidelines [32] confirmed by clinical examination and imaging (CT or MRI); (3) All patients with cerebral arteriovascular malformations and aneurysms; (4) South Asian or Caucasian ethnic backgrounds, and (5) Patient or relative written informed consent. Exclusion criteria include: (1) Unable to provide consent themselves or through surrogate, and; (2) stroke not defined on CT or MRI. Patients with old and recurrent stroke as well as seriously ill and/or intubated patients will also be included in the study in order to avoid bias against more severe forms of stroke.

### 2B2. Controls

Spouse/partners will be recruited as control subjects. As they tend to arise from the same geographical population and usually have similar exposure to environmental determinants, spouse/partner controls are suitable for case-control studies [33]. It is anticipated that occasionally spouses/partners will not be available. However, BRAINS is large enough to accommodate this. The use of unrelated and unaffected siblings could also act as suitable controls should there be any small shortfall [34]***. BRAINS-UK ***will recruit spouses or partners or unrelated unaffected relatives of South Asian ancestry as controls and ***BRAINS-SA ***will recruit 1500 spouses or partners or unrelated unaffected relatives as controls. Controls identified by probands will be asked to attend a hospital appointment for a direct venepunture procedure to obtain blood samples. If samples cannot be recruited in this way then packages for blood collection will be sent to controls identified either by the probands or their relatives. Letters requesting that they have blood extracted by their GP will accompany the packages. Controls will meet the following inclusion criteria: (1) should be aged > 18 years at the time of enrolment; (2) no previous history of stroke, and; (3) able to provide written consent. Spouse/partners will be identified on the inpatient acute stroke unit and stroke-free status will be confirmed using the BRAINS questionnaire. Hospitalized patients with any medical condition are not eligible as controls for BRAINS. Any lag in spouses as controls will be filled by recruiting age and sex matched community volunteers. Gender differences will be balanced by using large numbers of sex-mixed cases and/or sex-stratification during genetic analysis.

### 2C. Data collection

#### 2C1. Interview

Each participating stroke centre will have a developed stroke service and a stroke physician/neurologist/geriatrician with an interest in stroke. A detailed interview will be conducted by the local stroke trial coordinator/recruiter with each patient or their surrogate and controls to explain to them the aim of the study and role of their participation. Written consent will be required to be included in this study. Baseline clinical and demographic data such as clinical diagnosis, CT or MRI results, age, sex, ethnicity and other information will be collected. Proband reported medical and family history will also be recorded. Family history will not be verified independently by study coordinators. All details of the interview will be recorded on the BRAINS patient data collection form.

#### 2C2. Medical Records

Stroke research practitioners will review medical records of patients whose primary diagnosis is stroke before assessing eligibility for enrolment. Demographic, clinical and imaging / laboratory data will be collected on all patients using a standardised performa to ensure consistency of data across centres. The following information will be recorded on the case report forms: (1) Cardiovascular risk factors: vital signs (height, weight, blood pressure, and temperature), age, sex, ethnic origin, past history of hypertension and other cardiovascular disease, diabetes, smoking status, alcohol intake (U/wk), family history, (2) biomedical data: fasting glucose, lipids (total and HDL-Cholesterol and triglycerides), prothrombin time, Protein C and S, fibrinogen, plasma homocysteine concentration, lipoprotein analysis along with other biochemical markers, and; (3) imaging results: CT or MR of the head, size and location of the symptomatic cerebral infarct as seen on head imaging. Carotid Doppler will be undertaken when patients present with anterior circulation lesions. Emergency room blood pressure and biochemistry test results will be recorded.

#### 2C3. Stroke characterization

A study appointed neurologist/stroke physician will confirm a clinical diagnosis of stroke. The time of onset will be determined by talking to the patient and/or relatives present at the time of its occurrence. The neurologist will determine severity of neurological deficits within 48 hours of admission, based on the National Institute of Health Stroke Scale (NIHSS)[35]. Diagnosis of stroke will be confirmed using a CT scan or MRI of the brain, performed after the onset of symptoms. Post stroke functional status will be assessed using the Barthel Index[36] within 48 hours of admission. The TOAST classification system will be used to subtype stroke by a neurologist that is blinded to the genetic and phenotypic data.

#### 2C4. Patient follow-up

Patients are followed up by the study coordinators at the centre from where they were recruited. Patients will be telephoned after one year of recruitment to determine if they have had another vascular event. If patients are suffering from speech or cognition deficits, then history will be recorded from spouse or relative. In the event of mortality, cause of death will be ascertained.

#### 2C5. Genetic data

Trained personnel at each participating centre obtain 10 ml peripheral blood samples from patients and controls, in EDTA-coated vials using a single venepuncture. Each sample is assigned a unique BRAINS repository ID number and immediately stored at -20°C. Archive quality high-molecular weight genomic DNA is isolated from the peripheral lymphocytes using commercially available Qiagen DNA isolation kits. As a quality control, OD260/OD280 ratio is measured and accepted if above 1.8. For lower OD260/OD280 ratio's DNA samples will be repurified.

#### 2C6. Adverse events

All adverse events and other vascular events will be recorded by the study managers and forwarded to the principle investigator.

### 2D. Outcome measures

The primary outcome of the present study is to establish the largest DNA repository of highly phenotyped stroke patients of mainly South Asian descent. We aim to recruit 1500 stroke patients from BRAINS-UK and 3000 subjects (1500 cases) from BRAINS-SA. The secondary outcome is to identify novel gene loci associated with different types of stroke. Stroke patients will be compared with controls to determine the frequency and distribution of these susceptibility loci. Other important outcome measures include association of gene polymorphisms with stroke subtypes, ethnic groups and different environmental factors such as hypertension, diabetes and smoking.

### 2E.Data Analysis

BRAINS is a population-based case control study that will allow a hypothesis free genome-wide association (GWA) approach to identify genetic risk factors associated with stroke in European Caucasians and South Asians. Tests for Hardy-Weinberg equilibrium will be conducted separately for cases and controls. Genotypic analyses will consider allele, dominant, recessive and additive genetic models. Odds ratios will be determined using logistic regression analyses and the significance of association will be determined using the Chi-square or Fisher-exact test for allele, dominant and recessive genetic models.

### 2E1. Sample size and power

#### BRAINS-UK

All estimates of power are based on a meta-analysis of the ACE/ID polymorphism in stroke that showed a RR of 1.3 [37]. For the study as a whole we have ~ 1500 cases with a similar number of controls, giving us 90% power to detect a relative risk (RR) of 1.29 at p < 0.0001 with a population allele frequency of 0.2 (which would be appropriate for 100 candidate genes). For an allele frequency of 0.2 and a RR of 1.33 a more stringent p value of < 0.00001 is achieved at the same 90% power. For sub-group analysis of different stroke-types 430 cases and 430 controls allows us to detect a 1.5 RR with an allele frequency of 0.2 at a p-value of 0.01, with 80% power.

#### BRAINS-SA

We intend to enrol over 3000 subjects into the study over a 3 year period. Based on the current number of patients seen with radiologically confirmed ischemic stroke of 500 per annum this should be a readily achievable target by the Indian and Sri Lankan arms within the specified time frame. This stand alone case-control study of 1500 cases and 1500 controls provides ~90% power for an OR of 1.3 at p < 0.0001. These numbers broadly hold true for genome wide association studies using the Illumina platforms [38]. The power of this study not only depends on the frequency of the 'risk allele' but also on the relative risk conferred by this allele and on the chosen type-1 and -2 errors. However, this study is powered to detect clinically significant gene effects. Moreover, BRAINS can accommodate future advances in single nucleotide polymorphism (SNP) technology such as second generation whole genome sequencing [39].

## 3. Discussion

Stroke is a major public health burden in the UK costing the National Health Service (NHS) about £7 billion per year in direct care costs, loss of productivity, disability and informal costs [40]. The disease ranks as the second largest cause of mortality and disability in the UK after heart disease and cancer [41,42]. Stroke affects about 130,000 people in England and Wales alone with a higher incidence risk in people older than 75 years of age [43]. The majority (69%) of all strokes are ischemic whereas 19% are haemorrhagic in nature. Of the latter, 13% are classified as intracerebral haemorrhage and 6% subarachnoid haemorrhage. However, 12% of all strokes have an uncertain diagnosis [44]. Worldwide, stroke ranks as the third largest cause of death after ischemic heart disease and cancer and accounts for 3% of the world's disability burden [45]. The USA alone, reports 795,000 new and recurrent strokes per year [46]. India, China and Russia collectively account for the highest number of stroke cases in the world, with 80% of deaths occurring in 2005 in India from stroke alone [47]. It is estimated that by 2050, 80% of the global burden of new strokes of 15 million will occur in the low and middle income countries such as India[48,49].

The BRAINS study is a dedicated genetic risk association study with well classified inclusion and exclusion criteria which allow recruitment of large numbers of stroke patients and controls. The detailed repository of information and DNA bank allows a unique opportunity to apply both candidate gene and whole-genome approaches to cerebrovascular disease. The latter aim will be realised by increasing availability of SNP databases and gene chips. The use of spouses as controls allows candidate genes hypotheses to be tested quickly using the method of allelic-association, while controlling for environmental variability. BRAINS allows both established and novel candidate genes (acting either independently or with other risk genes) to be detected, which in time, may facilitate identification of novel potential therapeutic targets. This is particularly important for a disease like stroke where there are few treatment options.

Sustainability and the development of a long-term collaboration is a major objective to this initiative and once the biobank is fully established it can be used as a permanent international genetic resource to spawn new ideas and answer important questions in stroke genetics, particularly those relating to genetic differences of effects as a result of differing ancestral origins. BRAINS is uniquely placed as the largest repository for South Asian stroke data consisting of samples from Northern, Central and Southern South Asia. The results from our study will contribute to ongoing GWAS studies in stroke in understanding the genetic and molecular underpinnings of stroke.

## 4. List of Abbreviations

BRAINS: Bio-Repository of DNA in stroke; CT: Computed Tomography; MRI: Magnetic resonance imaging; NIHSS: National Institutes of Health Stroke Scale; GWAS: Genome Wide Association studies; TOAST: Trial of ORG10172 in Acute Stroke Treatment; UK: United Kingdom; SA: South Asia; MTHFR: Methylenetetrahydrofolate reductase; FVL: Factor V Leiden; ACE: Angiotensin-converting enzyme; PT: Prothrombin

## 5. Competing interests

The authors declare that they have no competing interests.

## 6. Authors contribution

SY is a research postgraduate at Imperial College London and has co-written the final protocol. RS, and MSK are BRAINS-UK project managers in London. JS is a stroke specialist nurse and research postgraduate at Imperial College London who contributed to developing BRAINS. AM is the BRAINS-SA project manager in New Delhi. Professors KP and PShr are the New Delhi site investigators who contributed to developing the final local protocol. Dr RdS is the Sri Lankan site investigator. Dr. PB is a Neurologist who contributed to the TOAST classification of probands. Professor PF provides genomics expertise and Professor JK cardiovascular expertise. Dr. PSha is the principal investigator of BRAINS (UK and SA) and has developed the protocol from its inception to the final version.

## Pre-publication history

The pre-publication history for this paper can be accessed here:

http://www.biomedcentral.com/1471-2350/12/34/prepub

## References

[B1] World congress of cardiology (WCC) scientific sessions, Beijing, China 2010 http://www.World-heart-federation.Org/congress-and-events/world-congress-of-cardiology-scientific-sessions-2010/press/press-releases/detail/article/stroke-incidence-and-mortality-rates-found-to-be-higher-in-developing-than-in-developed-countries-fo/accessed on 20.12.2010

[B2] Meschia JF Brott TG Brown RD Jr Crook RJ Frankel M Hardy J Merino JG Rich SS Silliman S Worrall BB The ischemic stroke genetics study (ISGS) protocol BMC Neurol 2003 3 4 10.1186/1471-2377-3-4 12848902 PMC184375

[B3] Meschia JF Brown RD Jr Brott TG Chukwudelunzu FE Hardy J Rich SS The siblings with ischemic stroke study (SWISS) protocol BMC Med Genet 2002 3 1 10.1186/1471-2350-3-1 11882254 PMC79001

[B4] Marchini J Cardon LR Phillips MS Donnelly P The effects of human population structure on large genetic association studies Nat Genet 2004 36 512 517 10.1038/ng1337 15052271

[B5] Reich D Thangaraj K Patterson N Price AL Singh L Reconstructing Indian population history Nature 2009 461 489 494 10.1038/nature08365 19779445 PMC2842210

[B6] Stewart JA Dundas R Howard RS Rudd AG Wolfe CD Ethnic differences in incidence of stroke: Prospective study with stroke register BMJ 1999 318 967 971 10195965 10.1136/bmj.318.7189.967 PMC27822

[B7] Gretarsdottir S Thorleifsson G Reynisdottir ST Manolescu A Jonsdottir S Jonsdottir T Gudmundsdottir T Bjarnadottir SM Einarsson OB Gudjonsdottir HM Hawkins M Gudmundsson G Gudmundsdottir H Andrason H Gudmundsdottir AS Sigurdardottir M Chou TT Nahmias J Goss S Sveinbjornsdottir S Valdimarsson EM Jakobsson F Agnarsson U Gudnason V Thorgeirsson G Fingerle J Gurney M Gudbjartsson D Frigge ML Kong A Stefansson K Gulcher JR The gene encoding phosphodiesterase 4d confers risk of ischemic stroke Nat Genet 2003 35 131 138 10.1038/ng1245 14517540

[B8] Staton JM Sayer MS Hankey GJ Attia J Thakkinstian A Yi Q Cole VJ Baker R Eikelboom JW Association between phosphodiesterase 4d gene and ischaemic stroke J Neurol Neurosurg Psychiatry 2006 77 1067 1069 10.1136/jnnp.2006.092106 16914755 PMC2077747

[B9] Matsushita T Kubo M Yonemoto K Ninomiya T Ashikawa K Liang B Hata J Doi Y Kitazono T Ibayashi S Iida M Kiyohara Y Nakamura Y Lack of association between variations of PDE4D and ischemic stroke in the Japanese population Stroke 2009 40 1245 1251 10.1161/STROKEAHA.108.527408 19246712

[B10] Hsieh M-S Phosphodiesterase 4d (PDE4D) gene variants and risk of ischemic stroke in the Taiwanese population LABMEDICINE 40

[B11] Anand SS Yusuf S Vuksan V Devanesen S Teo KK Montague PA Kelemen L Yi C Lonn E Gerstein H Hegele RA McQueen M Differences in risk factors, atherosclerosis, and cardiovascular disease between ethnic groups in canada: The study of health assessment and risk in ethnic groups (share) Lancet 2000 356 279 284 10.1016/S0140-6736(00)02502-2 11071182

[B12] Banerjee I Gupta V Ganesh S Association of gene polymorphism with genetic susceptibility to stroke in Asian populations: A meta-analysis J Hum Genet 2007 52 205 219 10.1007/s10038-006-0098-x 17171228

[B13] Williams B Poulter NR Brown MJ Davis M McInnes GT Potter JF Sever PS Mc GTS Guidelines for management of hypertension: Report of the fourth working party of the british hypertension society, 2004-BHS IV J Hum Hypertens 2004 18 139 185 10.1038/sj.jhh.1001683 14973512

[B14] Hurley D Begin treating hypertension sooner in blacks Cardiovascular Medicine, The Annual Meeting of the American Society of Hypertension 2010 21

[B15] O'Donnell MJ Xavier D Liu L Zhang H Chin SL Rao-Melacini P Rangarajan S Islam S Pais P McQueen MJ Mondo C Damasceno A Lopez-Jaramillo P Hankey GJ Dans AL Yusoff K Truelsen T Diener HC Sacco RL Ryglewicz D Czlonkowska A Weimar C Wang X Yusuf S Risk factors for ischaemic and intracerebral haemorrhagic stroke in 22 countries (the interstroke study): A case-control study Lancet 376 112 123 20561675 10.1016/S0140-6736(10)60834-3

[B16] Seshadri S Beiser A Pikula A Himali JJ Kelly-Hayes M Debette S DeStefano AL Romero JR Kase CS Wolf PA Parental occurrence of stroke and risk of stroke in their children: The Framingham study Circulation 121 1304 1312 10.1161/CIRCULATIONAHA.109.854240 20212282 PMC2860311

[B17] Mvundura M Family history as a risk factor for early-onset stroke/transient ischemic attack among adults in the United States Public Health Genomics 2010 13 13 20 10.1159/000209879 19307734

[B18] De Silva DA Woon FP Chen CL Chang HM Wong MC Ethnic South Asian ischaemic stroke patients have a higher prevalence of a family history of vascular disease compared to age, gender and diabetes-matched ethnic chinese subjects J Neurol Sci 2009 285 118 120 10.1016/j.jns.2009.06.013 19573878

[B19] Alberts MJ McCarron MO Hoffmann KL Graffagnino C Familial clustering of intracerebral hemorrhage: A prospective study in North Carolina Neuroepidemiology 2002 21 18 21 10.1159/000048609 11744821

[B20] Bentley P Peck G Smeeth L Whittaker J Sharma P Causal relationship of susceptibility genes to ischemic stroke: Comparison to ischemic heart disease and biochemical determinants PLoS One 2010 5 e9136 10.1371/journal.pone.0009136 20161734 PMC2817726

[B21] Hamedani AG Cole JW Mitchell BD Kittner SJ Meta-analysis of Factor V Leiden and ischemic stroke in young adults: The importance of case ascertainment Stroke 2010 41 1599 1603 10.1161/STROKEAHA.110.581256 20616326 PMC3180852

[B22] Peck G Smeeth L Whittaker J Casas JP Hingorani A Sharma P The genetics of primary haemorrhagic stroke, subarachnoid haemorrhage and ruptured intracranial aneurysms in adults PLoS One 2008 3 e3691 10.1371/journal.pone.0003691 19008959 PMC2579487

[B23] Ariyaratnam R Casas JP Whittaker J Smeeth L Hingorani AD Sharma P Genetics of ischaemic stroke among persons of non-european descent: A meta-analysis of eight genes involving approximately 32,500 individuals PLoS Med 2007 4 e131 10.1371/journal.pmed.0040131 17455988 PMC1876409

[B24] Marjot T Genes associated with adult cerebral venous thrombosis Stroke 2011 in press 21350198 10.1161/STROKEAHA.110.602672

[B25] Roberts R Ryan TJ Task force 3: Clinical research in a molecular era and the need to expand its ethical imperatives J Am Coll Cardiol 1998 31 942 949 10.1016/S0735-1097(98)80182-4 9561991

[B26] The ad hoc committee on genetic testing/insurance issues: Genetic testing and insurance Am J Hum Genet 1995 56 327 331 7825594 PMC1801295

[B27] Foster M Naming names in human genetic variation research (editorial) Genome Research 1998 8 755 757 9724320 10.1101/gr.8.8.755

[B28] Guidelines for human biobanks and genetic research databases - consultation on the draft guidelines Medical Research Council (MRC) 2008

[B29] Adams HP Jr Bendixen BH Kappelle LJ Biller J Love BB Gordon DL Marsh EE Classification of subtype of acute ischemic stroke. Definitions for use in a multicenter clinical trial. Toast. Trial of org 10172 in acute stroke treatment Stroke 1993 24 35 41 7678184 10.1161/01.str.24.1.35

[B30] Adams HP Jr Brott TG Crowell RM Furlan AJ Gomez CR Grotta J Helgason CM Marler JR Woolson RF Zivin JA Guidelines for the management of patients with acute ischemic stroke. A statement for healthcare professionals from a special writing group of the stroke council, american heart association Stroke 1994 25 1901 1914 8073477 10.1161/01.str.25.9.1901

[B31] Adams H Guidelines for thrombolytic therapy for acute stroke: A supplement to the guidelines for the management of patients with acute ischemic stroke. A statement for healthcare professionals from a special writing group of the stroke council Stroke 1996 27 1711 1718 8784157

[B32] Who monica project principal investigators World Health Organization Monica project (monitoring trends and determinants in cardiovascular disease) A major international collaboration Journal of Clinical Epidemiology 1988 41 105 114 10.1016/0895-4356(88)90084-4 3335877

[B33] Worrall BB Chen DT Meschia JF Ethical and methodological issues in pedigree stroke research Stroke 2001 32 1242 1249 11387482 10.1161/01.str.32.6.1242 PMC2613840

[B34] Curtis D Use of siblings as controls in case-control association studies Ann Hum Genet 1997 61 319 333 10.1017/S000348009700626X 9365785

[B35] Brott T Adams HP Jr Olinger CP Marler JR Barsan WG Biller J Spilker J Holleran R Eberle R Hertzberg V Measurements of acute cerebral infarction: A clinical examination scale Stroke 1989 20 864 870 2749846 10.1161/01.str.20.7.864

[B36] Mahoney FIBD Functional evaluation: The barthel index Maryland State Med Journal 1965 14 56 61 14258950

[B37] Sharma P Meta-analysis of the ace gene in ischaemic stroke J Neurol Neurosurg Psychiatry 1998 64 227 230 10.1136/jnnp.64.2.227 9489536 PMC2169971

[B38] Amos CI Wu X Broderick P Gorlov IP Gu J Eisen T Dong Q Zhang Q Gu X Vijayakrishnan J Sullivan K Matakidou A Wang Y Mills G Doheny K Tsai YY Chen WV Shete S Spitz MR Houlston RS Genome-wide association scan of tag snps identifies a susceptibility locus for lung cancer at 15q25.1 Nat Genet 2008 40 616 622 10.1038/ng.109 18385676 PMC2713680

[B39] Mardis ER Next-generation DNA sequencing methods Annu Rev Genomics Hum Genet 2008 9 387 402 10.1146/annurev.genom.9.081307.164359 18576944

[B40] Reducing brain damage: Faster access to better stroke care National Audit Office Report, Department of Health 2005

[B41] Wolfe C The burden of stroke Stroke Services and Research 1996

[B42] Beswick J Stroke and disability Journal of Stroke and Cerebrovascular Diseases 2004 13 10.1016/j.jstrokecerebrovasdis.2004.06.003 17903971

[B43] Stroke incidence and risk factors in a population-based cohort study Office of National Statistics, Health Statistics Quarterly 2001 12

[B44] Stroke statistics 2006 The Stroke Association

[B45] Warlow C Stroke The Lancet 2003 362 1211 1224 10.1016/S0140-6736(03)14544-8 14568745

[B46] Lloyd-Jones D Heart disease and stroke statistics--2010 update: A report from the american heart association Circulation 2010 121 46 215 10.1161/CIRCULATIONAHA.109.192667 20019324

[B47] Who chronic diseases report 2005

[B48] Murray CJL Alternative projections of mortality and disability by cause 1990-2020: Global burden of disease study Lancet 1997 349 1498 1504 10.1016/S0140-6736(96)07492-2 9167458

[B49] Feigin VL Stroke in developing countries: Can the epidemic be stopped and outcomes improved? Lancet Neurology 2007 6 94 97 10.1016/S1474-4422(07)70007-8 17239789

